# Factor structure of intelligence and divergent thinking subtests: A registered report

**DOI:** 10.1371/journal.pone.0251268

**Published:** 2021-05-12

**Authors:** Russell T. Warne, Sam Golightly, Makai Black

**Affiliations:** Department of Behavioral Science, Utah Valley University, Orem, Utah, United States of America; University of Bologna, ITALY

## Abstract

Psychologists have investigated creativity for 70 years, and it is now seen as being an important construct, both scientifically and because of its practical value to society. However, several fundamental unresolved problems persist, including a suitable definition of creativity and the ability of psychometric tests to measure divergent thinking—an important component of creativity—in a way that aligns with theory. It is this latter point that this registered report is designed to address. We propose to administer two divergent thinking tests (the verbal and figural versions of the Torrance Tests of Creative Thinking; TTCT) with an intelligence test (the International Cognitive Ability Resource test; ICAR). We will then subject the subscores from these tests to confirmatory factor analysis to test which of nine theoretically plausible models best fits the data. When this study is completed, we hope to better understand whether the degree to which the TTCT and ICAR measure distinct constructs. This study will be conducted in accordance with all open science practices, including pre-registration, open data and syntax, and open materials (with the exception of copyrighted and confidential test stimuli).

## Introduction

In 1950, J. P. Guilford gave his presidential address to the American Psychological Association, calling for psychologists to produce more research on creativity. Ever since Guilford’s [1] address, the topic has been one of the most valued among educational and differential psychologists. Creativity in the sciences and the arts is an engine for economic and cultural progress [2], and important in its own right as a construct.

One of Guilford’s [1] concerns when he encouraged more research on creativity was that psychology’s emphasis on analytical intelligence in testing and research made scholars neglect creativity. In Guilford’s view, “creativity and creative productivity extend well beyond the domain of intelligence,” [1, p. 445], and intelligence tests were unable to measure creative methods of problem solving. Guilford theorized over the ensuring decades until his death about the relationship between intelligence and creativity. By the end of his life, he had incorporated creative thinking functions into his sprawling Structure of Intellect Model [3] as one of the operations humans perform while solving cognitive tasks [4].

Another topic of Guilford’s [1] landmark address is the relationship with intelligence. He believed that the two constructs were undoubtedly positively correlated. Studies designed to analyze the relationship between creativity and intelligence have consistently produced only modest correlations, often in the range of *r* = .10 to .30 [5–9]. One of the seminal studies on creativity and intelligence was Wallach and Kogan’s [10] analysis of 151 5th graders. The children were given multiple creativity and intelligence tests. Scores from the tests within each group intercorrelated well with one another (*r* = .41 between creativity tests, and *r* = .51 between intelligence tests), but the correlation between these categories was low (*r* = .09; [10]), providing strong evidence of divergent validity. Silvia [5] later used latent variable analysis and replicated Wallach and Kogan’s [10] results, giving strong evidence for a consistent yet marginal relationship between creativity and intelligence. In a similar publication, Plucker [11] reanalyzed creative achievement data from Torrance’s [12] longitudinal study. Using structural equation modeling, he found that divergent thinking scores predict creative achievement with much stronger predictive validity than intelligence scores, and concluded that, divergent thinking and intelligence “represent relatively independent constructs” [11, p. 111].

This finding is sometimes disputed, though. Methodological and assessment artifacts make the strength of the correlation between intelligence and creativity scores appear artificially weaker.[13] Silvia [9] found that latent variable correlations tend to be stronger than observed variable correlations, and that sample size, task type, and the number of responses can influence the apparent correlation between intelligence and creativity test scores. Studies based on the Berlin Structure of Intelligence model seem to indicate that some cognitive abilities that contribute to intelligence have moderate or strong relationships with divergent thinking and/or creativity, including mental speed [14], working memory [15, 16], and associative fluency [15]. This may indicate the correlation between intelligence and creativity test scores is due to an overlap in at least some cognitive abilities that contribute to behaviors that are labeled “intelligent” or “creative.” And in the domain of mathematical creativity, the correlation with intelligence test scores is often stronger than the correlation between general creativity measures (like divergent thinking scores) and intelligence [7].

The mere presence of a positive correlation between creativity and intelligence test scores is not enough to establish the nature of the relationship between the two constructs. A variety of theoretical and causal models could produce the positive correlations so frequently found between measures of intelligence and creativity [6]. Before discussing these models, it is important to explore the definitions of both creativity and intelligence.

## Definitions of creativity

Theories of creativity reach back thousands of years. Acknowledging differences in thought processes was present in the culture of Ancient Greece [17], though people also “believed that creativity required the intervention of the muses” [18, p. 152], and artists were not considered creative in and of themselves. The earliest empirical inquiry into human creative behavior can be traced to Galton’s [19] work on human capabilities. In more recent decades, defining creativity has been perhaps the most central topic and challenge in contemporary creativity research [20]. Guilford [1] was an early champion of the importance of scientific research in creativity, yet even he seemed to struggle defining what creativity is; “In its narrow sense, creativity refers to the abilities that are most characteristic of creative people” (p. 444). Runco and Jaeger [20] contended that the first clear scholarly description of creativity was offered by Stein [21], who defined creativity as, “a novel work that is accepted as tenable or useful or satisfying by a group in some point in time” (p. 311). Stein’s [21] definition has been adopted by many researchers [22–24] and includes two important criteria: originality and usefulness.

Originality refers to an idea or product not having previously existed. This is crucial to the construct of creativity. The ability to convincingly forge the Mona Lisa may require talent, but it does not necessarily mean a person is creative. Creativity requires novelty. However, many “ideas and products that are merely original might very well be useless” [20, p. 93], and so novelty is a necessary but not sufficient qualification for creativity. Therefore, for products to qualify as creative, they must be original *and* useful.

The dual criteria of originality and usefulness have been troublesome for creativity researchers, primarily because judging originality and usefulness involves some subjectivity [25, 26]. The novelty required for a product to qualify as creative could theoretically be measured objectively, but this would require accurately determining whether a product has previously existed somewhere in the world at some point in time. To prove that an idea is truly novel and has never existed before is inherently illogical because it requires proving a negative. Usefulness is even more subjective, because it relies almost entirely on context. This usefulness is often determined by the community or group that the product is created for or by individuals who encounter the idea or product. Runco et al. [27] postulated that from this perspective, “creativity can lead to things which are original and useful but only for the individual creator himself or herself” [27, p. 366].

Although Stein’s [21] requirements that creative thought results in original and useful products, others have expanded theories of creativity to include other behaviors and characteristics. Another important facet of creativity is the influence of non-cognitive elements like personality and motivation on its expression [22]. For example, Furnham and Bachtiar [28] and Furnham and Nederstrom [29] found that personality trait extroversion correlated positively with test scores of divergent thinking. Similarly, Wang et al. [30] found that extroversion correlated substantially with scholarly creative achievement in undergraduate students. Openness to experience has also been shown to relate to creative expression. Benedek, et al. [31] found that jazz musicians scored higher on tests of divergent thinking and openness to experience compared to their folk and classically trained counterparts. Fink and Woschnjak [32] found a similar pattern in dancers; modern/contemporary performers scored higher in creativity and openness to experience than individuals trained in theater and ballet. In addition to the direct relationship between openness and creativity, openness mediates the relationships between temperament variables and creativity [33]. Temperament can also directly influence creative expression. For example, Nęcka and Hlawacz [34] found the temperament trait activity correlated positively with creativity while emotional reactivity was negatively correlated. In other words, people with active and emotionally nonreactive temperaments scored higher on creativity tests.

Motivation also likely influences creative expression. Hannam and Narayan [35] found that university students were more likely to produce creative work if they were intrinsically motivated and perceived the work environment as fair. Saether [36] also discovered that motivation mediated the relationship between creativity and fairness in a sample of online survey responses. These results seem in line with Silvia et al.’s [37] assertion that motivation and its effect on creativity includes aspects of, “goals, self-regulatory processes, and experiences that foster or impede wanting to invest time in creative activities” (p. 114).

Moreover, it is possible that many individuals possess the capacity to be creative but choose not to do so [23]. Torrance acknowledged that scores from creativity tests do not ensure creative accomplishment [27]. Because of its multifaceted nature, with roots in personality, the social environment, and cognition, Mumford and Gustavson [38] have argued that creativity ought to be considered the product of a system of characteristics, rather than a single, isolatable characteristic.

Thus, creativity is a complex, multifaceted construct. However, there are commonalities among the dueling definitions. Weiss et al. [13] found that across definitions idea generation (often called fluency) and originality are part of nearly every scholarly definition of creativity. These two aspects of creative behavior are well captured in the concept of divergent thinking, which is the focus of our scholarly investigation.

### Importance of creativity

Creativity represents one of the pinnacles of human experience. Indeed, it is difficult to imagine human progress in any of its forms without the catalyzing spark and sustaining force of creativity. Vygotsky held that, “creativity is an essential condition for existence and all that goes beyond the rut of routine” [39, p. 11]. This alone makes creativity worthy of research, but some theorists have proposed other reasons.

One reason to empirically study creativity is its role in fostering economic growth. There is a major economic need for domestic and international industries to quickly and efficiently solve problems, and many simple jobs that need little to no creative output are being automated [40]. It will become increasingly important for the advancing global economy to foster and identify creativity in all age groups [38]. Creativity is crucial in educational organizations as well. In a review of the empirical creativity literature, Davies et al. [41] found evidence that creative learning environments positively impacted student academic attainment, concentration, enjoyment, enthusiasm, and emotional development.

### Measuring creativity

An essential requirement for the empirical study of creativity is a method of measuring creative behavior. The most widely used measures of creativity are the Torrance Tests of Creative Thinking (TTCT), which consists of a Verbal test reliant on written linguistic responses and a non-verbal Figural test that uses pictorial stimuli and requires responses that the examinee must draw (see detailed description below). However, the TTCT does not measure the totality of creative thinking; instead, it measures one of the building blocks of creative behavior: divergent thinking, i.e., the capacity to produce a variety of ideas in response to a stimulus [42]. The TTCT measures divergent thinking through fluency (i.e., the number of responses generated), originality (i.e., how much the responses differ from common responses given in the norm sample), and flexibility (which is the variety of types of responses that examinees give). Divergent thinking has been shown to positively correlate with individual creative achievement [27] and is so important to creativity that some researchers claim that divergent thinking is the most valid way to predict creativity, almost using the two terms interchangeably [43]. However, others deny these claims and hold that divergent thinking is only a predictor of individual creativity [22, 44].

Despite the popularity of the TTCT, there remain uncertainties about some of the test’s psychometric properties. One of the questions regarding the TTCT is the dimensionality and factor structure of its scores. Multiple studies have suggested that tests of divergent thinking are multidimensional and follow a two-factor model [45–49]. The authors of each of these studies termed these two factors innovative and adaptive, patterned after Kirton’s [50] Adaptor-Innovator theory (KAI). The KAI postulates problem-solving and creativity are often manifested in one of two styles; the adaptive style is characterized by doing things better, while the innovator style is characterized by doing things differently [50]. In each of these studies fluency and originality loaded onto the innovative factor and elaboration and abstractness of title loaded onto the adaptive factor [45–49]. Resistance to premature closure had less consistent results, loading onto both the innovative and adaptive factors in [45] and [48], just the innovative factor in [47], and just the adaptive factor in Humble et al. [46] and [49].

If the KAI theory is correct, it would present a problem for TTCT because the tests’ subscores and global score do not align with the theory. Instead of two subscores—innovation and adaptation—each version of the TTCT produces a global score and three (for the TTCT-V) or five (for the TTCT-F) subscores. This represents a major deficit in the internal validity evidence of the TTCT and its ability to give test users a correct understanding of examinees’ divergent thinking. Because this study is psychometric in nature, it will not provide definite answers to the question of how creativity and intelligence relate to one another as constructs. Instead, this study is limited to investigating the degree to which divergent thinking and intelligence subtests form separate latent factors.

## Defining intelligence

Like most constructs in the social sciences, there are many definitions of intelligence (see [51] for a compilation of definitions from leading theorists). Since the mid-1990s, though, one conceptual definition has found widespread—though not unanimous—agreement among experts in intelligence:

Intelligence is a very general mental capability that, among other things, involves the ability to reason, plan, solve problems, think abstractly, comprehend complex ideas, learn quickly and learn from experience. It is not merely book learning, a narrow academic skill, or test-taking smarts. Rather, it reflects a broader and deeper capability for comprehending our surroundings—“catching on,” “making sense” of things, or “figuring out” what to do. [52, p. 13]

Most definitions overlap with this one, often encompassing a global ability to engage in problem solving (e.g., [53]) or learn from one’s environment and/or experience (e.g., [54]), though some theorists and researchers have proposed definitions that are neurological in origin (e.g., [55]) or culturally specific (e.g., [56]), or that extend beyond cognitive abilities (e.g., [57]). Alternative definitions, however, have not found widespread acceptance, and most psychologists still consider complex reasoning and general cognitive competence as some of the central components of intelligence.

In addition to verbal definitions, many psychologists subscribe to a statistical definition, where intelligence is taken as being similar or equivalent to a general factor that statistically captures approximately half of variance in the scores on a set of cognitive tasks. This factor is often called *g* or Spearman’s *g* (in honor of its discoverer). Unlike a verbal definition, the statistical definition of intelligence is much less subjective or ambiguous. Moreover, because of the indifference of the indicator, the *g* factor that emerges from different intelligence tests are nearly identical (with factor correlations often *r* = .95 or higher), indicating that *g* as a statistical definition of intelligence is not dependent on any particular test or collection of tasks [58–64]. In this paper, we will subscribe to the statistical definition and use a common factor of scores on cognitive tasks as our operationalization of intelligence.

## Investigating the relationship between divergent thinking subtests and intelligence subtests via factor structure

### Factor structure of intelligence tests

In contrast with the TTCT, the factor structure of scores from intelligence tests is well established. Almost all cognitive test batteries produce a series of scores that either load onto a single factor or produce a number of factors that, in turn, load onto a single general factor [64, 65]. This general factor has been called *g* since Spearman [66] discovered it over a century ago. Among psychologists studying intelligence, *g* is a mainstream theory, and there is strong evidence that every cognitive task loads on *g* to some extent [63]. Spearman [67, pp. 197–198] called this phenomenon the “indifference of the indicator,” and it has led some experts to argue that every task in life that requires cognitive work is its own intelligence test, which would explain why IQ scores correlate with so many life outcomes [68–70].

The indifference of the indicator presents a major challenge to the widely accepted belief that creativity tests—especially the TTCT with its focus on divergent thinking—represent a distinct construct. TTCT test content is unquestionably cognitive, and according to the theory of the indifference of the indicator, TTCT content should measure *g*, at least partially. Moreover, in the past, some researchers designing tests to measure other constructs have accidentally created tests that measured intelligence [63, Chapter 7]. For example, Sanders et al. [71] found that the Defining Issues Test, a test designed to measure moral development and reasoning, is *g*-loaded and is a moderately good measure of verbal intelligence. Likewise, literacy tests are highly *g* loaded and—in American samples—do not seem to measure a construct that is distinct from intelligence [72]. These examples and the strong evidence in favor of the indifference of the indicator raise the possibility that the TTCT is actually a measure of intelligence. The best way to investigate this possibility is through psychometric study of the factor structure of scores from the TTCT and an intelligence test when given to the same sample of examinees.

The factor structures of both the TTCT and intelligence tests have been studied independently, but we have been unable to identify any research examining the factor structure of creativity and intelligence tests at the same time to determine whether these tests measure the same latent construct, or multiple constructs (and how multiple constructs might be related to one another). Our goal in conducting this study is to determine the factor structure of subtests drawn from intelligence and divergent thinking tests when administered together. Through this study we aim to determine the degree to which divergent thinking subtests and intelligence subtests measures separate constructs. We intend to use confirmatory factor analysis to determine which of several plausible factor structures provides the best fit for the subtest data.

### Theoretically plausible factor structures

Sternberg and O’Hara [73] described five possible ways that could describe the relationship between intelligence and creativity:

- Creativity is a component of intelligence.
- Intelligence is a component of creativity.
- Creativity and intelligence are different constructs with overlapping components and/or mental processes.
- Creativity and intelligence are different labels for what are, substantially, the same problem solving construct.
- Creativity and intelligence are separate constructs with any correlations between the two being incidental.

Sternberg and O’Hara [73] found that there was evidence supporting all of these viewpoints, making it hard to distinguish which one is the best description of the relationship between intelligence and creativity. Years later, Karwowski et al. [6] added another possibility: that intelligence is a necessary but not sufficient condition for high creativity, which may explain the positive correlations between the two but also why many high-IQ individuals fail to engage in creative behaviors. Complicating the picture is that the relationship between the two constructs may be dependent on the context [74], with a degree of domain knowledge often being necessary for a person to generate creative ideas [2].

With the exact nature of the relationship between intelligence and creativity remaining an unresolved question, we posit that the positive correlation between the two constructs may be partially due to a measurement artifact that some tasks that measure creativity may be—like all other cognitive tasks—partially measuring intelligence. To investigate this possibility, we propose this study to determine whether the divergent thinking tasks on the TTCT are measures of general intelligence (i.e., are *g*-loaded).

The possibility that divergent thinking tests also measure intelligence is not far-fetched. Over 100 years ago, Binet and Simon [75, pp. 229–230] included a task on his second intelligence test in which a child examinee was asked to name as many words as possible in three minutes. Although the task was scored quantitively by tallying up the total number of words that the examinee produced, Binet discussed the qualitative differences in responses among children, noting the different categories of words that some examinees generated or the uniqueness of their responses. Modern creativity researchers would recognize Binet’s quantitative scoring procedure as a measure of fluency and his qualitative analysis as touching upon originality and flexibility in responses [42]. More recently, Carroll [65] identified fluency as manifestation of a broad mental retrieval ability that was subsumed by a general intelligence factor. Other researchers from the psychometric tradition have also suggested that common measures of creativity and/or divergent thinking could be measuring aspects of intelligence [13].

From a psychometric perspective, there are several factor structures that we believe are plausible for explaining the relationship between scores on divergent thinking and intelligence tests: (a) distinct but correlated constructs, (b) scores loading directly on a *g* factor, and (c) a hierarchical structure with *g* as a second-order factor. We will briefly describe each of these possibilities here and then describe exact models we will use in our study in the Methods section.

Possibility (a), which is a factor structure of correlated separate constructs, would emerge if intelligence and divergent thinking subtests produce separate factors. This is the factor structure suggested by divergent validity research showing that intelligence and divergent thinking tests do not strongly correlate with one another (e.g., [11]). In the most straightforward form, this could occur if the TTCT and intelligence tests each produced their own factor(s) which were, in turn, correlated with one another. Structure (b) would occur if subscores from the divergent thinking subtests and intelligence tests all formed a single, undifferentiated general factor. This would support the intelligence community’s belief that all cognitive tasks measure *g* to some extent and be strong evidence that Spearman’s [67] theory of the indifference of the indicator is correct. Finally, possibility (c) would be a hybrid model of (a) and (b) where a number of factors can combine to form a single second-order *g* factor. This type of structure would support intelligence theorists’ belief in the universality of *g* but also permit the existence of first-order factors—such as divergent thinking and intelligence factors that span a number of subscores. In this study, we will assess 1–4 models of each type in order to better understand how intelligence test scores and divergent thinking test scores are related.

## Methods

### Ethics

The study received ethical approval from the Utah Valley University Institutional Review Board, protocol #441. Informed consent will be obtained at two time points: when examinees take the ICAR online and again at the beginning of the in-person testing session (when the TTCT tests are administered). The initial informed consent will be recorded online, while the informed consent obtained at the beginning of the in-person testing session will be obtained through a signed written consent document.

### Instruments

We will administer a total of thirteen subscores drawn from three professionally developed psychometric tests: the Torrance Tests of Creative Thinking Figural Test A, the Torrance Tests of Creative Thinking Verbal Test A, and an abbreviated version of the International Cognitive Ability Resource (ICAR) test.

#### TTCT figural test A

The TTCT Figural Test A consists of a picture construction activity, picture completion activity, and lines activity. The test is designed to measure divergent thinking with standardized pictorial stimuli.

The scoring system produces six subscores:

- fluency (defined as the capacity to produce a large number of visual images),
- originality (the production of unusual responses),
- elaboration (the capacity to create responses that are more embellished than a basic figure),
- abstractness of titles (the capability of producing non-literal titles for pictures),
- resistance to premature closure (generating responses that leave stimuli open-ended and do not close them immediately and prematurely), and
- a checklist of creative strengths.

The first five subscores are norm-referenced, and examinees receive points based on the degree to which their responses are more creative than those generated by the test’s norm sample. The checklist of creative strengths subscore is created by summing thirteen criterion-referenced scores that correspond to components of the examinees’ constructed responses on the three subtests [76].

The TTCT Figural Test A subtests will be administered with the time limits indicated in the test manual. However, we modified the instructions slightly because they seemed to be written for children, and our examinees will be adults. These modifications are minor in nature and are designed to remove language that we found overly simplistic or condescending. Altered instructions are available from the project’s page on the osf.io web site.

#### TTCT verbal test A

Like the TTCT Figural Test A, the TTCT Verbal Test A is designed to measure divergent thinking in a standardized fashion. The TTCT Verbal Test A consists of three ask-and-guess tasks, a product improvement task, an unusual uses subtask, and a just suppose task. These six tasks generate three subscores: fluency, flexibility, and originality. Fluency and flexibility are the same as for the TTCT Figural Test A. For the TTCT Verbal Test A, the flexibility subscore measures examinee’s adeptness at generating responses in different categories.

Just as with the TTCT Figural A test, the only modification we made to this test was to alter the instructions to better suit an adult examinee population. Altered instructions are available from the project’s page on the osf.io web site.

#### ICAR test

The ICAR test was developed as a free test of general cognitive ability that is available in the public domain for researchers to use [77]. The ICAR test consists of four subtests: verbal reasoning, 3D rotation, letter and number series, and matrix reasoning. The verbal reasoning subtest consists of items that use written English to ask questions about general knowledge, basic reasoning, and the relationships among words (e.g., synonyms, antonyms). The 3D rotation subtest shows a two-dimensional representation of a cube showing three sides. The examinee must then identify which of six options depicts a rotated version of the target cube. Examinees also have the option of indicating that none of the options is correct and another option to state that they do not know the answer. The letter and number subtest consists of a sequence of five letters or numbers (never both in the same test item), which the examinee must complete. Finally, the matrix reasoning subtest shows a 3 x 3 grid of geometric figures with one section replaced with a question mark. The examinee should select which of six options would complete the pattern shown in the matrix. The items on the ICAR test are typical for written intelligence tests. All item types have appeared on intelligence tests for at least 50 years and are well established methods for measuring intelligence in examinees [78].

To ensure we could give both TTCT tests and the ICAR test to our volunteer examinees in the allotted time, we were forced to make some adjustments to the ICAR test. The first was that we shortened the ICAR test from 60 items to 52 items: 16 items on the verbal reasoning subtest, 16 items on the 3D rotation subtest, 9 items on the letter and number series subtests, and 11 items on the matrix reasoning subtest. Using internal consistency reliability values reported by Condon and Revelle [77, Table 3], these shortened subtests are expected to have Cronbach’s α values of at least .70, based on estimates calculated from the Spearman-Brown prophecy formula.

Additionally, we have added time limits to each shortened subtest and allotted examinees 11 minutes to complete the verbal reasoning subtest, 16 minutes to complete the 3D rotation subtest, 8 minutes to complete the letter and number series, and 13 minutes to complete the matrix reasoning subtest. We determined these time limits by administering the original ICAR test untimed to a convenience sample of college students and young adults. Subtests that took longer than desired to complete were shortened, and items were dropped quasi-randomly to ensure that any subtest’s eliminated items varied in difficulty. The shortened ICAR test was pilot tested with more examinees with time limits based on the expected amount of time per item calculated from the untimed administrations of the full ICAR test. Further pilot testing of the abbreviated ICAR test indicated that most examinees will be able to attempt almost every item on the shortened version of the test.

### Data and scoring

For all three tests, we will follow test manual instructions for scoring items and tasks. Therefore, for both TCTT tests, we used subscores that had *not* been converted to standardized scores or age or grade percentiles. For the ICAR test, the raw scores were the number of test items correctly answered on each subtest. Data from examinees who leave the testing setting early and/or do not attempt all three tests will not be used in the analyses.

The scoring system for the TTCT Verbal and Figural tests is based on counts of valid responses (fluency), which are then used to produce scores for unusual responses (originality) and the number of categories responses fit into (elaboration). The TTCT Figural also uses counts to produce scores for abstract titles, resistance to closure, and the creative strengths checklist. Because achieving a high subscore on originality, elaboration, and any of the other scores is dependent on first generating a number of responses, these scores are confounded with the test’s fluency scores. Over the years, researchers have proposed different methods for handling this confounding, [42, 79] and there is no one clear correct method for doing so. The method we have chosen is to divide each task’s subscore by the fluency subscore for the same task, sum the subscores that measure the same aspect of divergent thinking, and to use these summed values in our analyses. This altered score can be interpreted as an average rate of divergent responses (i.e., original responses, elaborate responses, etc.) per valid response. Because there are other scoring alterations which can control for fluency, we will make our altered scores and the original scores available in our open data set so that researchers can use other methods for controlling for the confound of fluency and test our models (or other hypotheses) accordingly.

### Sample

Sample size will be set by funding constraints but will be large enough to meet guidelines for conducting confirmatory factor analysis studies. Our funding permits the purchase of test materials for 420 examinees. Assuming 5% data loss from incomplete tests or missing subscores, we will have enough test forms for 399 examinees. This will allow every model to have at least 20 sample members per estimated path. This exceeds the recommended minimum sample size for convergence and parameter estimate stability in confirmatory factor analysis [80]. Statistical power for the chi-squared difference tests cannot be calculated because realistic parameter estimates require realistic a priori estimates of all other model parameters [81], but because this is the first confirmatory factor analysis of both intelligence and creativity subscores, these estimates are not available.

The sample will consist of a convenience sample of students attending a large open-enrollment university in the western United States and members of the surrounding community. Students will receive course credit for participating in the testing session or for inviting a community member who participates. Psychology students at this university are required to participate in research as part of their education. Examinees will take the ICAR online (its typical administration format) up to one week before their in-person testing session. Total testing will last 120–150 minutes (including breaks between tests), with the TTCT tests being administered in person in a session that will last up to 120 minutes. TTCT test order will be randomized for each session.

### Analysis

We will report basic descriptive statistics for all variables: means and standard deviations for all subtest scores and demographic variables that are interval- or ratio-level data, along with frequency tables for nominal- and ordinal-level demographic variables. A correlation table will also be reported for all subtest scores.

Our plan is to use confirmatory factor analysis to examine nine plausible models for how the subscores on the TTCT tests and the ICAR test could relate to one another. These models are summarized briefly in Table 1. All confirmatory factor analyses will be performed with MPlus 8.4. We believe that a Registered Report fits our study design and analysis plan because “Registered Reports can be an empowering venue for testing new theories or arbitrating between competing theories” [82, p. 570]. Having locked-in and pre-registered models will remove any subjectivity from our study and ensure that the model selection process occurs without regard for our preference for any particular model.

**Table 1:** Proposed models for interrelationships among TTCT and ICAR subscores.

Model No.	Hierarchical?	Nested?	Reference Variable(s)	Notes
1a	No	Yes, within Models 2a and 5, and Model 4a is nested within this model	TTCT Figural Fluency, TTCT Verbal Fluency, ICAR Matrix Reasoning	Test-based model where the three tests form three correlated factors
2a	No	Yes, within Model 5, and Models 1a and 4a are nested within this model	TTCT Figural Fluency, ICAR Matrix Reasoning	Test-based model where the TTCT and ICAR form two correlated factors
3a	No	Yes, within Model 5	TTCT Figural Fluency, TTCT Verbal Fluency	Model with two correlated factors based on subtest stimuli; verbal subtests form one factor and non-verbal subtests form the second factor
4a	No	Yes, within Model 1a, which is nested within Model 2a, which is nested within Model 5	TTCT Figural Fluency, TTCT Closure Resistance, TTCT Verbal Fluency, and ICAR Matrix Reasoning	Model that splits the TTCT Figural factor in Model 2a into innovative and adaptive factors
5	No	Yes, Models 1a-4a are nested within this model (note that Model 4a is nested within Model 1a, which is nested within Model 2a, which is nested within Model 5)	ICAR Matrix Reasoning	Congeneric model with all subscores loading directly onto a single general factor
1b	Yes	No	TTCT Figural Fluency, TTCT Verbal Fluency, ICAR Matrix Reasoning, ICAR factor	Model 1a with a general factor subsuming the three test factors
2b	Yes	No	TTCT Figural Fluency, ICAR Matrix Reasoning, ICAR factor	Model 2a with a general factor subsuming the two test factors
3b	Yes	No	TTCT Figural Fluency, TTCT Verbal Fluency, Non-verbal factor	Model 3a with a general factor subsuming the verbal and non-verbal factors
4b	Yes	No	TTCT Figural Fluency, TTCT Closure Resistance, TTCT Verbal Fluency, and ICAR Matrix Reasoning	Model that splits the TTCT Figural factor in Model 2b into innovative and adaptive factors

The nine models fall into the three groups described in the introduction section and are all either hierarchical or non-hierarchical models. Models 1a, 2a, 3a, and 4a all represent a multifactor relationship between creativity and intelligence subscores where all factors are intercorrelated. Model 5 is a model where all scores load directly onto a general factor. Finally, the hybrid models are Models 1b, 2b, 3b, and 4b, which all have 2–4 initial factors which then load on a general factor, making them hierarchical models. All other models (i.e., 1a, 2a, 3a, 4a, and 5) are non-hierarchical models. Almost all non-hierarchical models have a corresponding hierarchical model with the same number of factors, but with the addition of a general second-order *g* factor. The exception to this is Model 5, which is a congeneric model where all subscores load directly onto a general factor.

**Fig 1:**
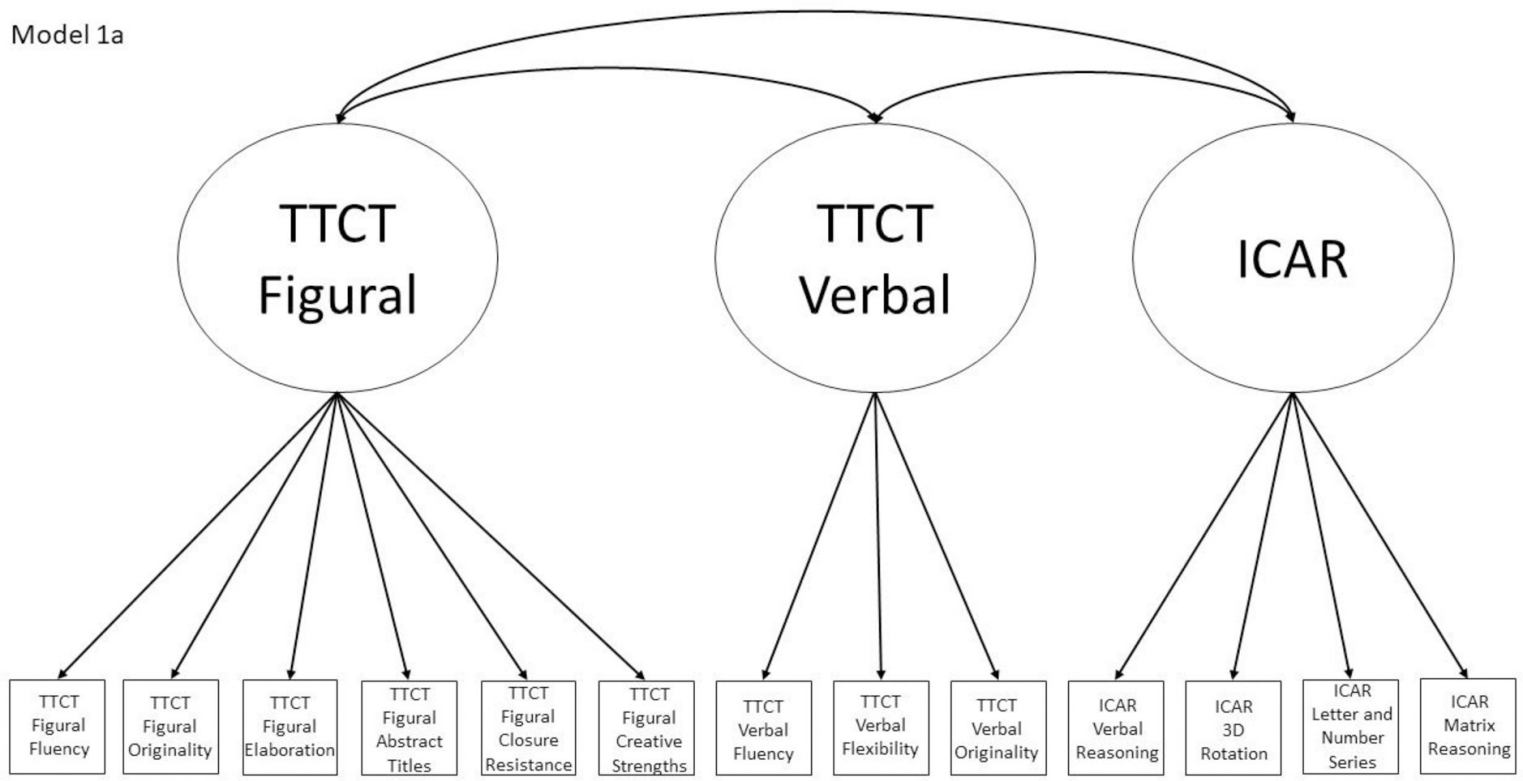
Confirmatory factor analysis diagram for Model 1a.

**Fig 2:**
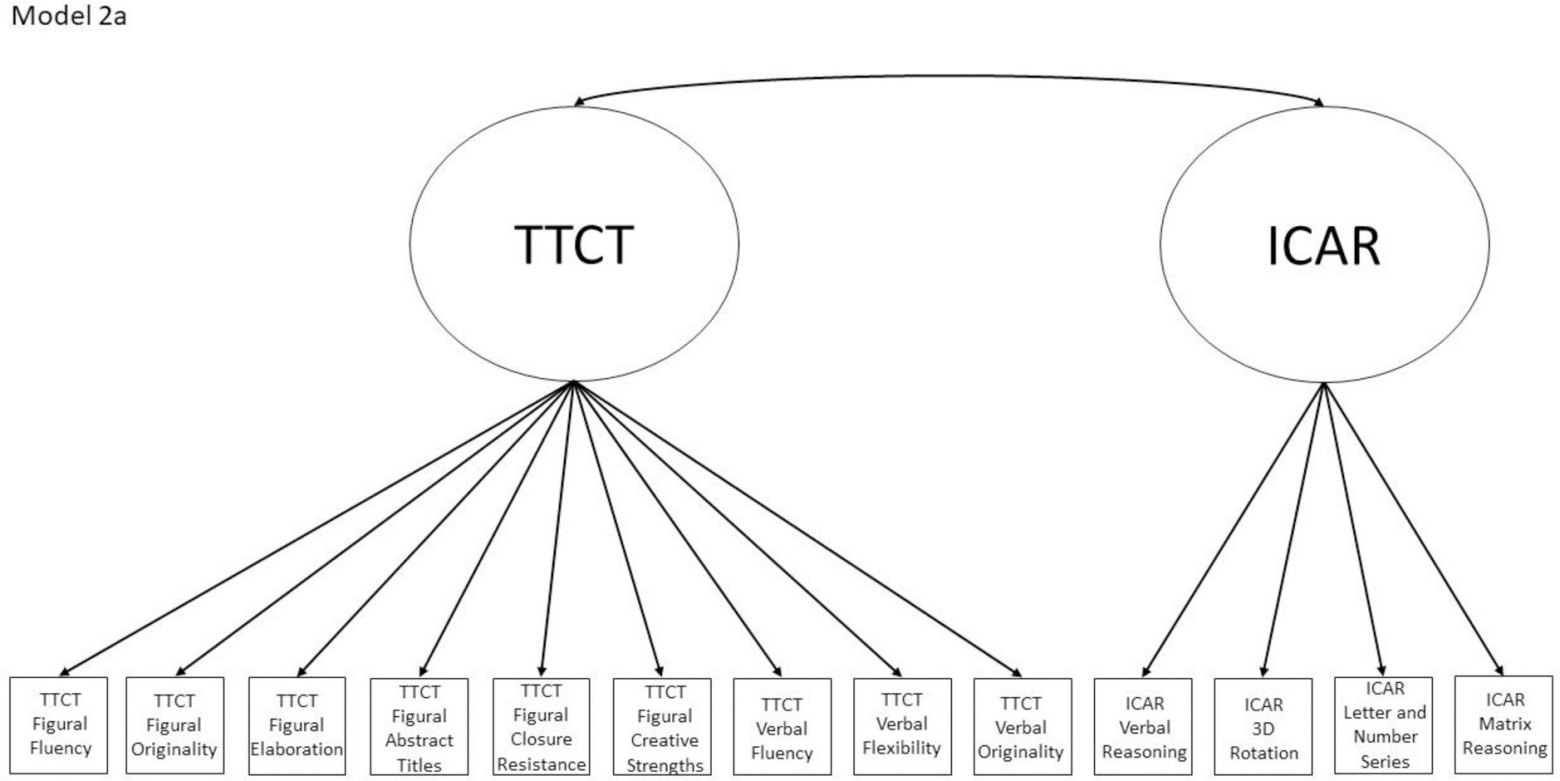
Confirmatory factor analysis diagram for Model 2a.

**Fig 3:**
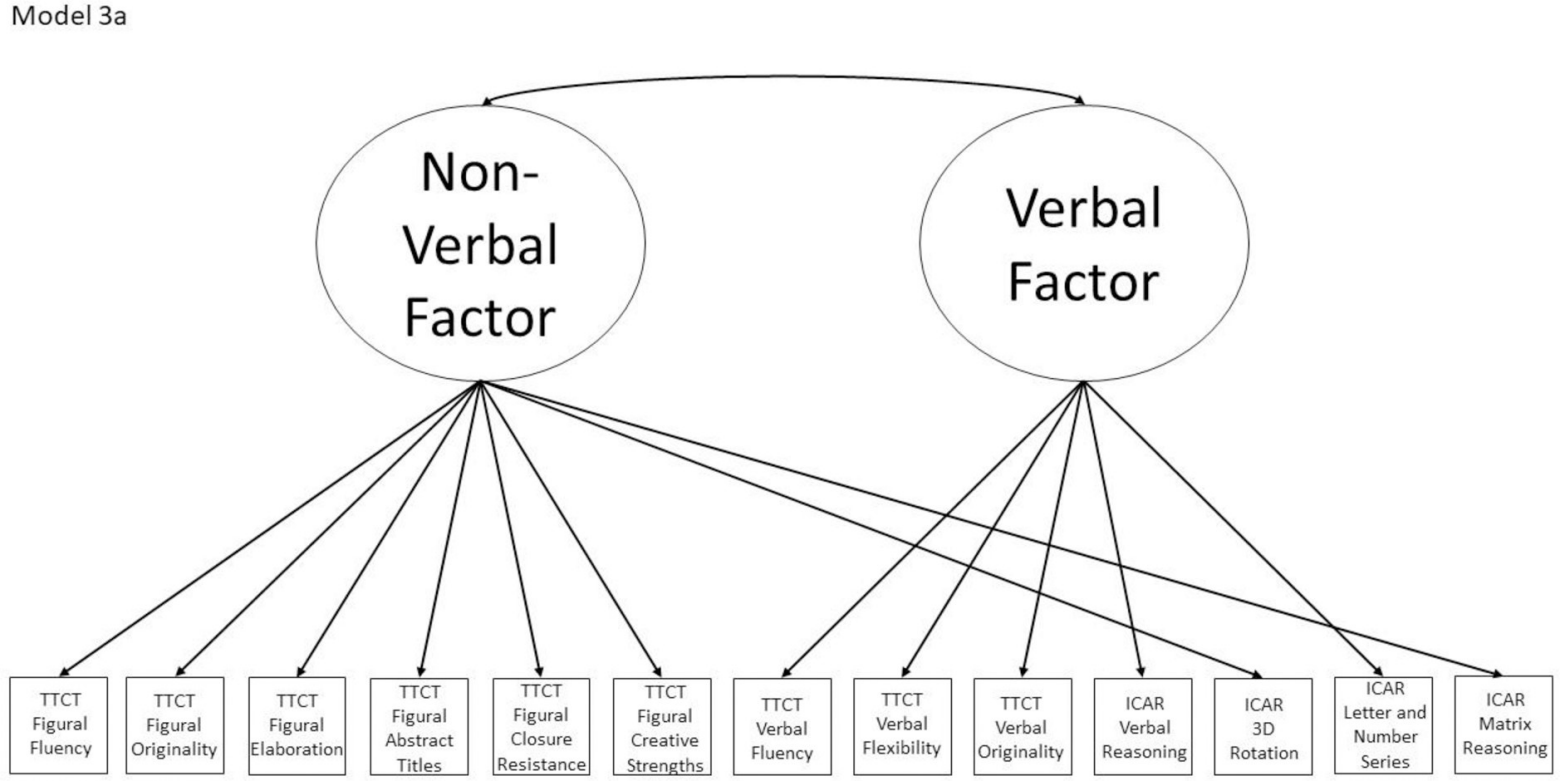
Confirmatory factor analysis diagram for Model 3a.

**Fig 4:**
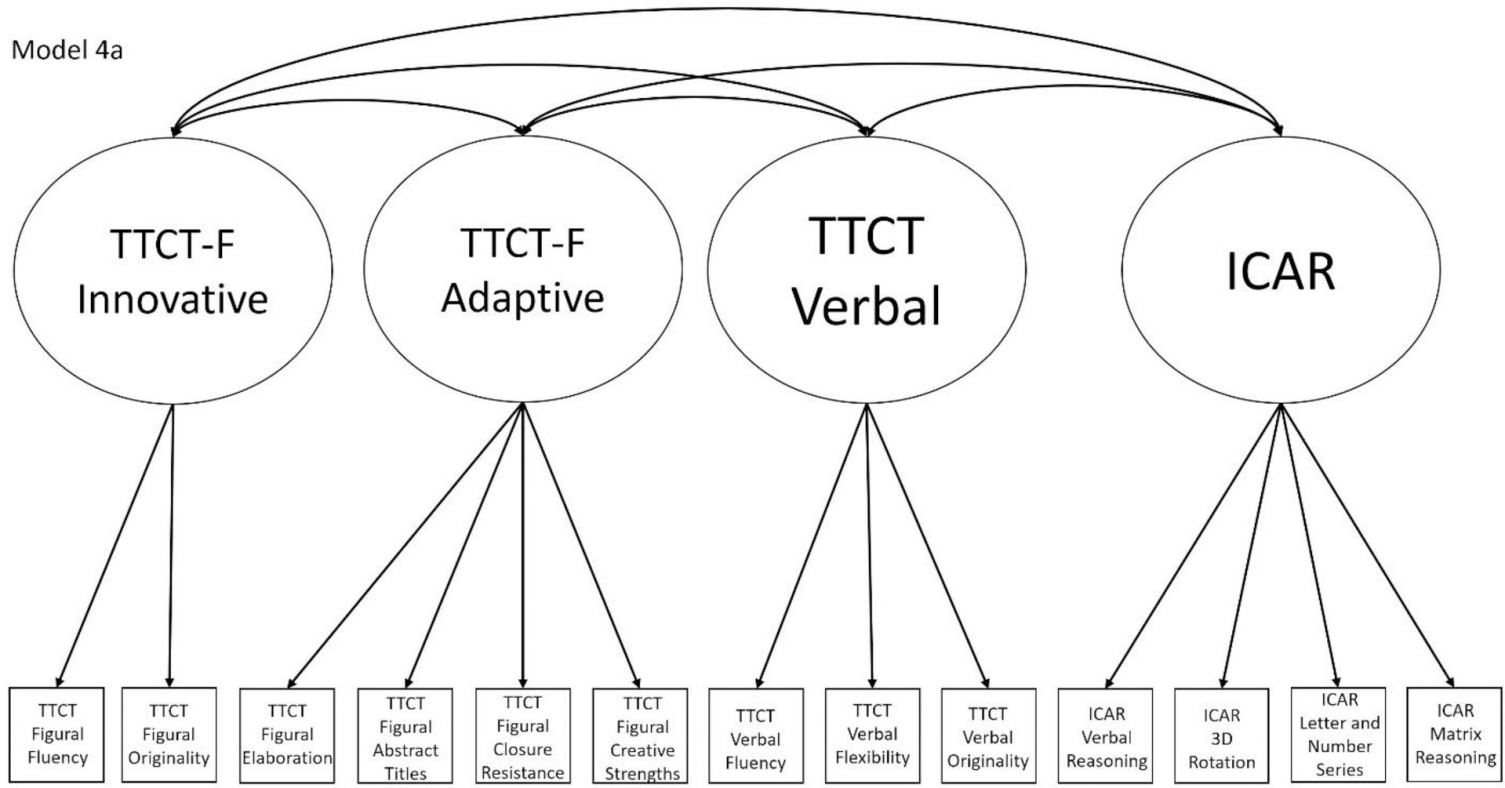
Confirmatory factor analysis diagram for Model 4a.

**Fig 5:**
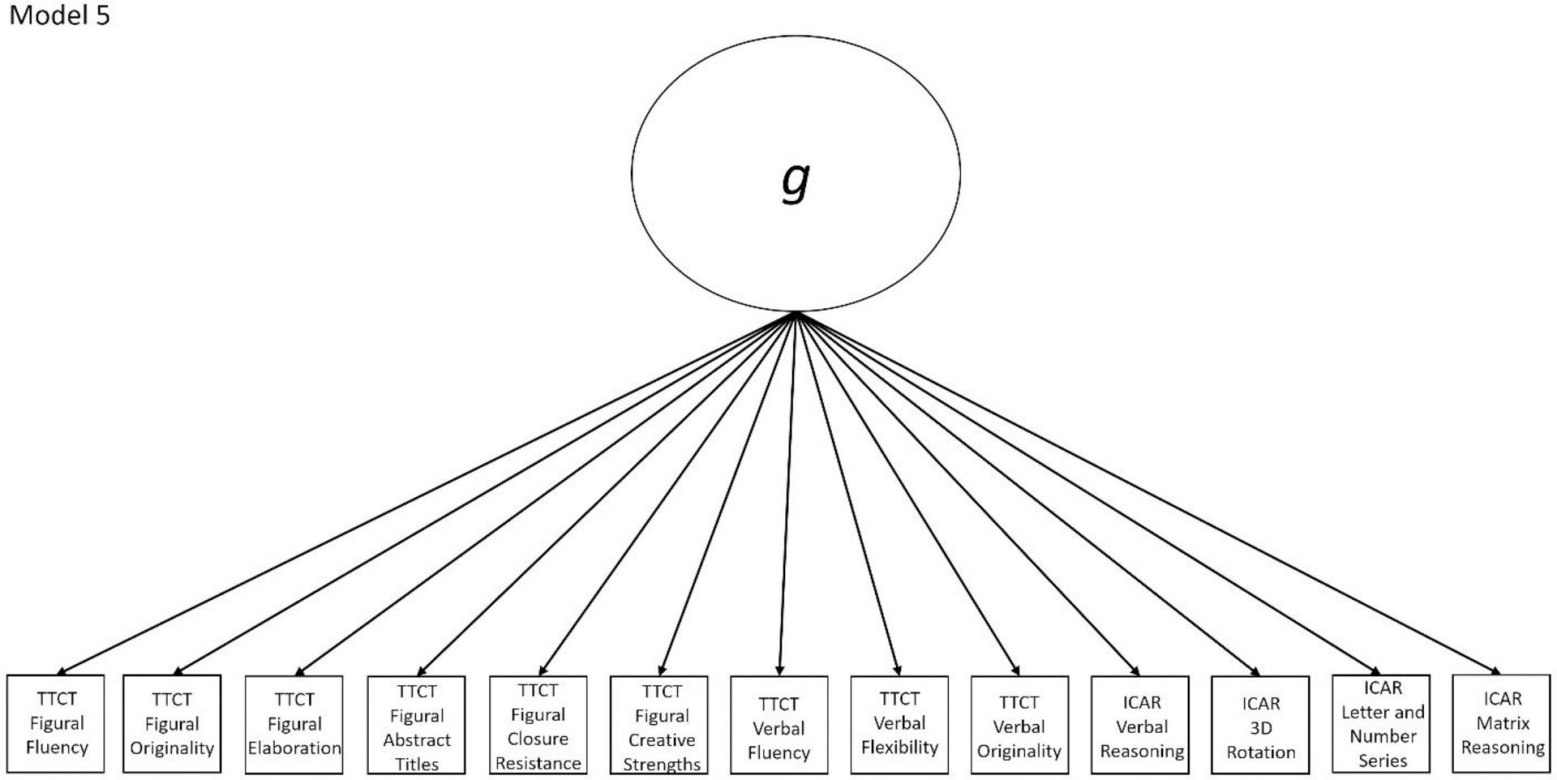
Confirmatory factor analysis diagram for Model 5.

**Fig 6:**
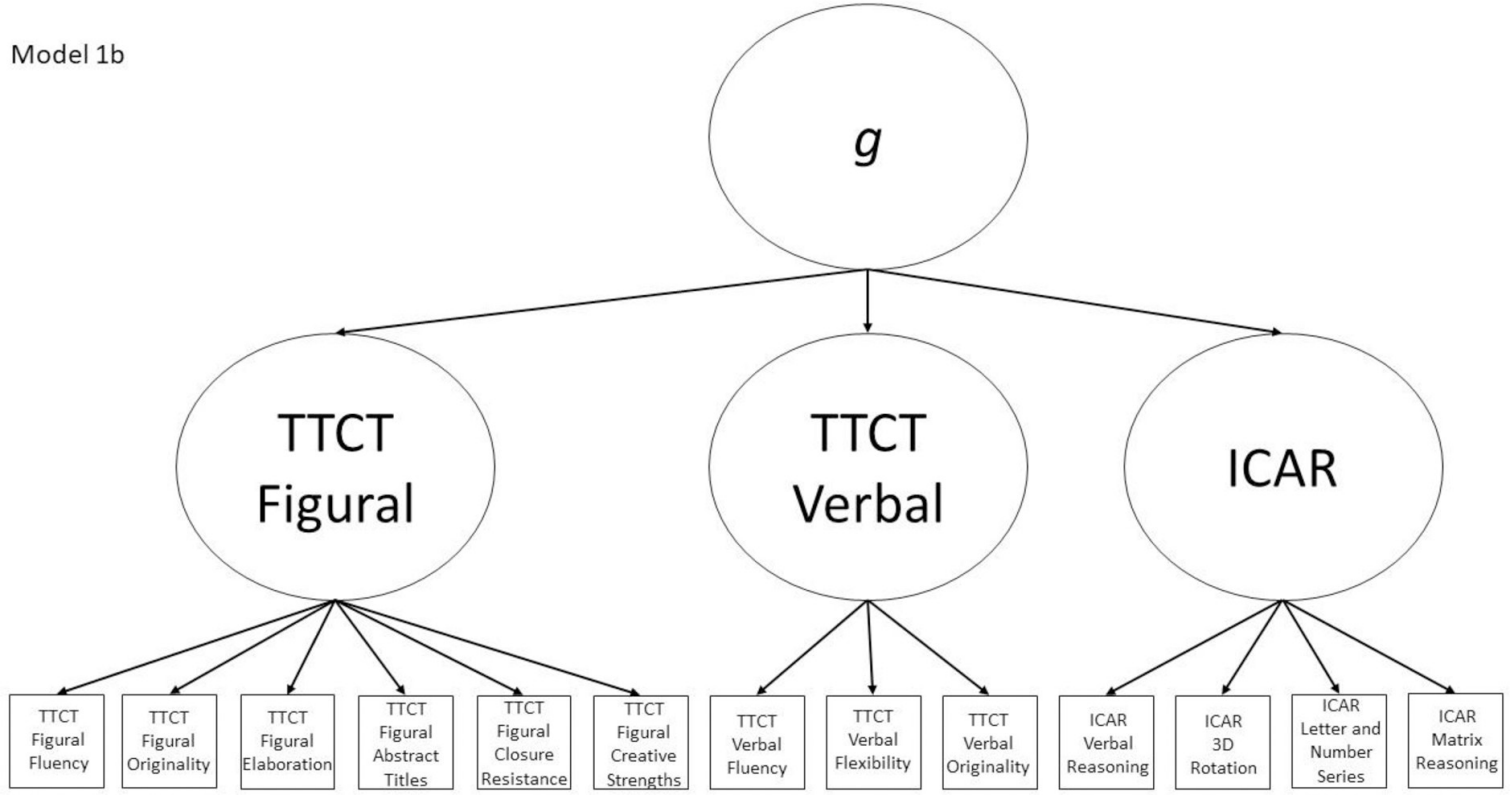
Confirmatory factor analysis diagram for Model 1b.

**Fig 7:**
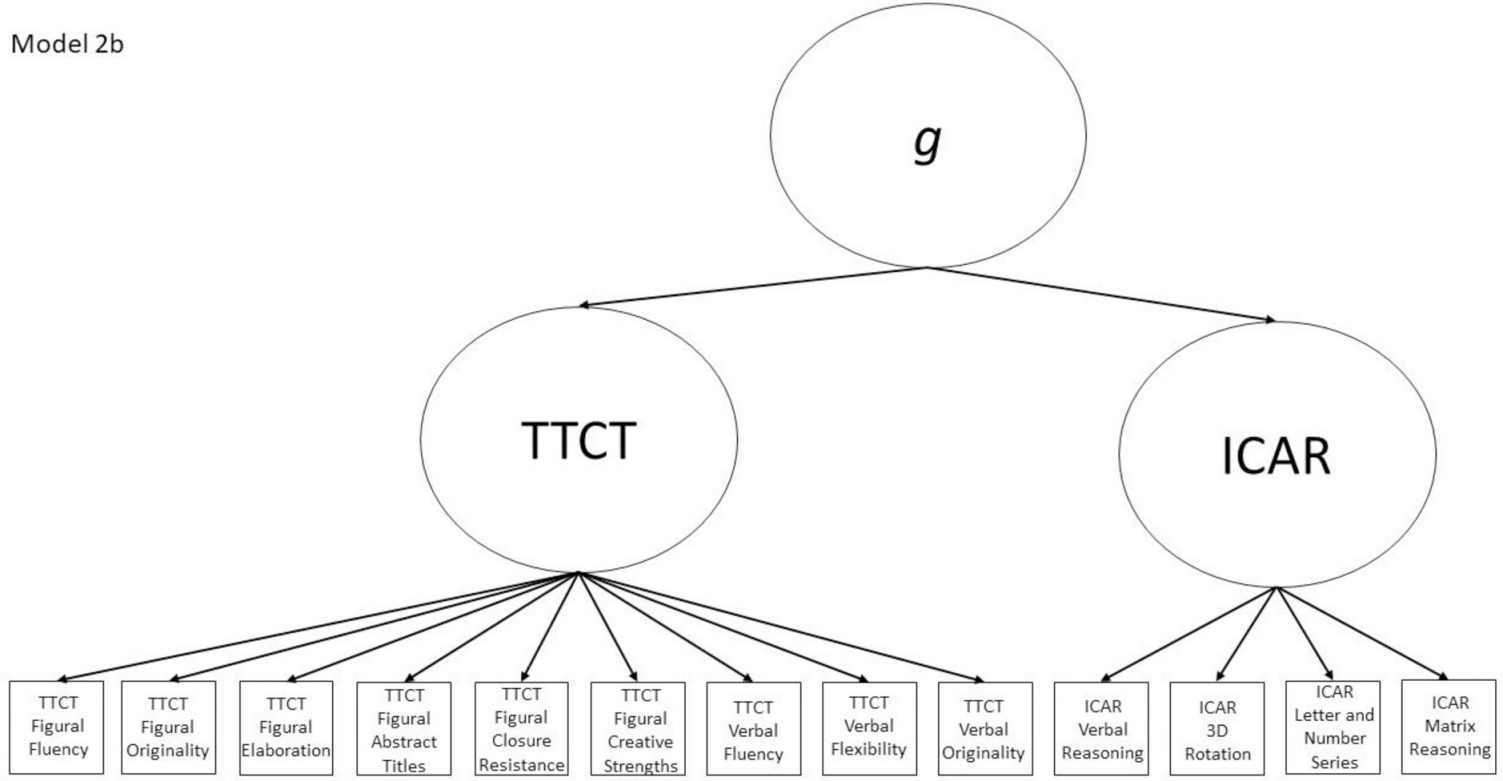
Confirmatory factor analysis diagram for Model 2b.

**Fig 8:**
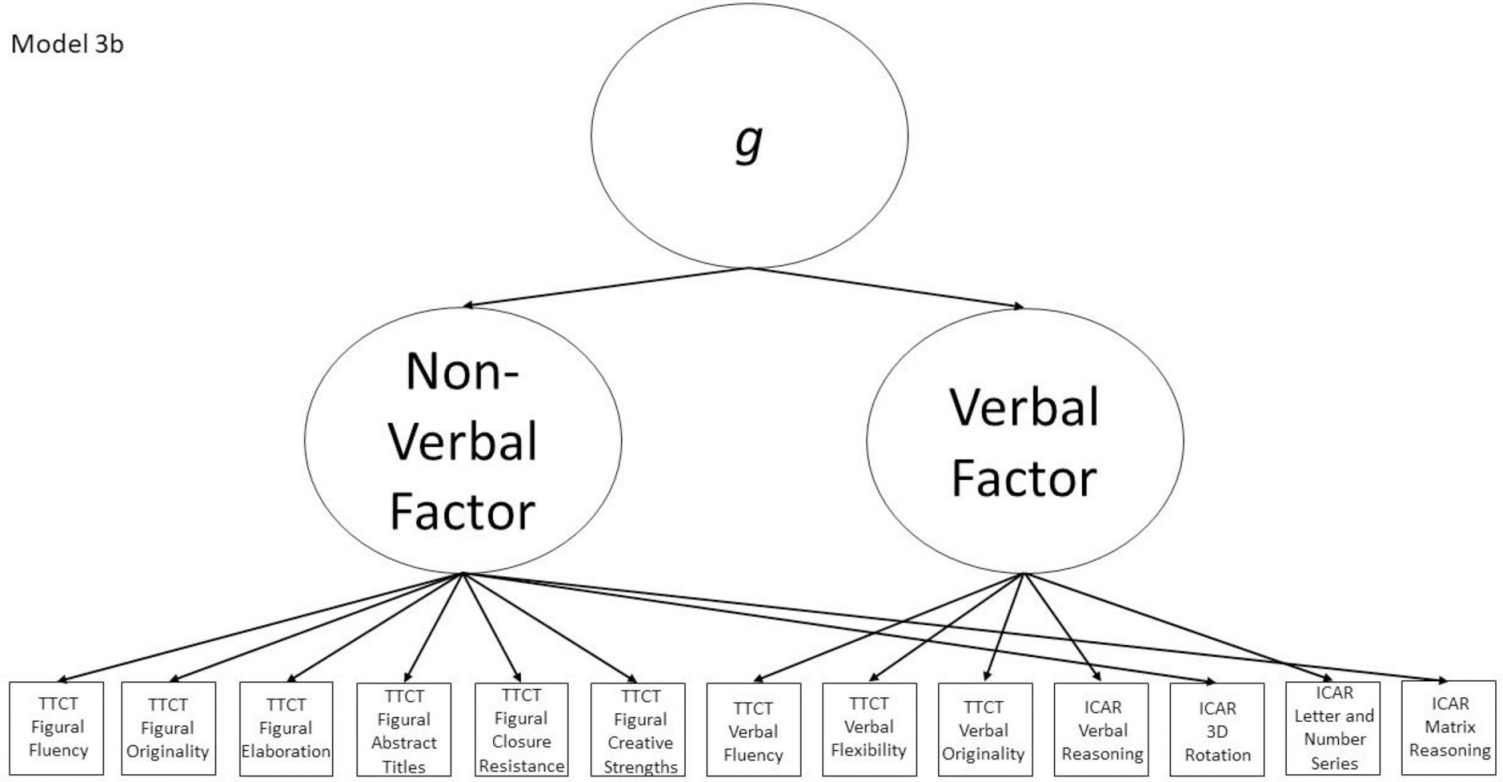
Confirmatory factor analysis diagram for Model 3b.

**Fig 9:**
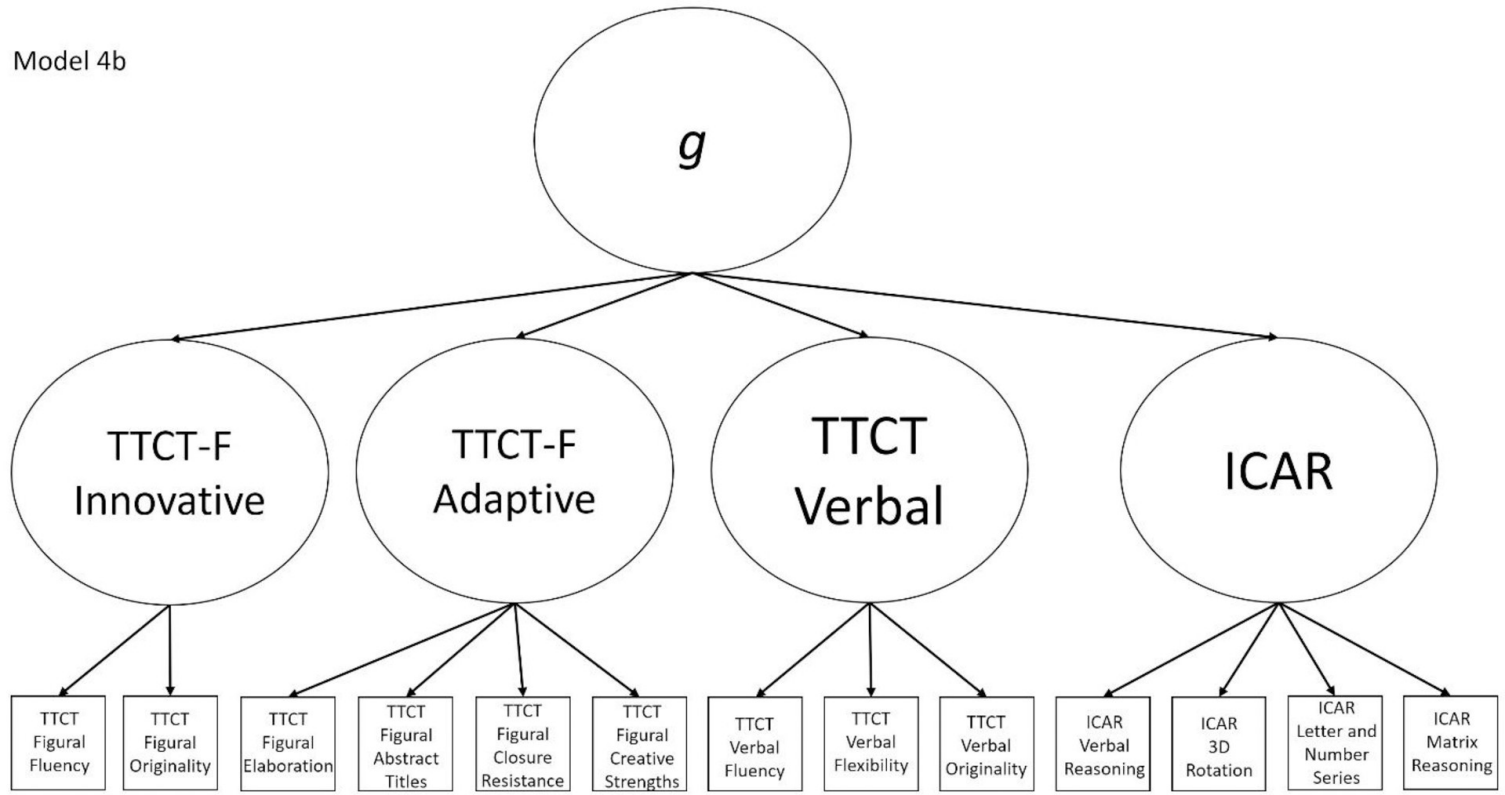
Confirmatory factor analysis diagram for Model 4b.

Models 1a and 1b have three first-order factors that are based on the tests, with both forms of the TTCT and the ICAR each forming its own factor based on its subscores. Models 2a and 2b are similar, but form two first-order factors: one for all TTCT subscores and another for ICAR subscores. These models would be appropriate if both forms of the TTCT measure divergent thinking and the ICAR measures a separate cognitive ability (i.e., intelligence). Models 3a and 3b have two second-order factors based on the subtest stimuli, with verbal scores all forming a factor and non-verbal scores forming a separate, correlated factor. These models would support the traditional dichotomization between these types of stimuli on intelligence tests, which dates back to David Wechsler. Models 4a and 4b are based on empirical evidence that subscores on the TTCT Figural test do not fit a one-factor model [47, 49] and instead are best represented with a two-factor model comprising of an “innovative factor” and an “adaptive” factor, which would support Kirton’s [50] adaptor-innovator theory. (Another measure of divergent thinking showed a similar two-factor structure; see [13]). All nine models are diagrammed in Figs 1–9. There will be no attempts to modify models in order to improve fit. However, because prior research shows that the creative strengths subscore on the TTCT Figural test can lead to poor model fit [47], we will also test these models without the creative strengths subscore. These results will be relegated to a supplemental file because the TTCT’s creator sees the creative strengths subscore as being essential for understanding a person’s performance on the TTCT Figural test [49].

Models will be identified with the reference variable strategy, where one variable’s factor loading is set to 1.0. The fluency scores and the matrix reasoning score will always be used for this purpose because these scores tend to have the strongest loadings on TTCT and intelligence factors in exploratory factor analyses [83–85]. For hierarchical models, the factor loading for the first-order ICAR factor on the second-order *g* factor will be set to 1.0 because if there is a general construct that parsimoniously explains performance on the subtests, then it is likely a general intelligence factor and should have a strong loading from a first-order intelligence factor.

### Interpretation

Fit statistics will be used to evaluate model fit and compare models with one another. We intend to use the chi-squared value, the comparative fit index (CFI), Tucker-Lewis Index (TLI), root mean square error of approximation (RMSEA) with 90% confidence interval, standardized root mean square residual (SRMR), Akaike information criterion (AIC), and Bayesian information criterion (BIC). These fit indices are suitable for making comparisons among competing models, and they are a cross-section of fit statistics with compensatory strengths and weaknesses [86, 87]. For this study, models will be judged to have acceptable fit if they have an SRMR value ≤ .08 and at least one of the following: (1) CFI ≥ .90, (2) TLI ≥ .90, or (3) RMSEA ≤ .08. These statistics will also be used to judge the best fitting model(s) among the nine. Models will be favored when their SRMR and RMSEA values are closer to zero and their CFI and TLI values are closer to 1.0.

The AIC and BIC will also be used to compare all models to one another, with lower values of these statistics indicating better model fit. When comparing models with the same number of degrees of freedom, both fit statistics will favor the same model, and the model with the lowest AIC and/or BIC will be preferred. When models have differing degrees of freedom, the penalty for a more complex model (i.e., with fewer degrees of freedom) will be more severe for the BIC than the AIC. Therefore, if a more complex model has a lower BIC than a simpler model, then it will be preferred because this would indicate that the model fits the data much better than the simpler model, despite the loss of parsimony.

Chi-squared difference tests will be used to compare nested models. Table 1 indicates which models are nested within one another. Model 5 is the most general of these models, with Models 1a, 2a, 3a, and 4a nested within it. These latter models will be compared to Model 5 in a sequential fashion by constraining a correlation between the relevant first-order factors to 1.0—in order to force these factors to merge—and conducting a chi-squared difference test with *k*—1 degree of freedom, where *k* is the number of factors being merged. There are two sequences of nested models that can be tested in this fashion. The first starts with the complex Model 4a, and testing whether it is statistically equal to Model 1a, followed by a test to determine whether Model 2a is statistically equal to Model 5. The second group of nested models will be to test whether Model 3a is statistically equal to Model 5 in the same fashion. All of these comparisons will be made, and any tests that produce a non-statistically significant result (with *p* > .05) will indicate that the two nested models are equivalent and that the simpler model with more degrees of freedom should be preferred. Note that to test nested models, we will constrain correlations between factors in the more complex model to be equal to 1.0 to create the more parsimonious model and test whether this model is statistically different from the more complex model. Because a constrained correlation of *r* = 1.0 creates a boundary constraint (because the correlation between factors cannot be greater than 1.0), we will use the appropriate mixed chi-squared distribution for each comparison (see [88] for details).

After identifying the best model(s) that fit the data, we will interpret the results in light of the different plausible relationships that divergent thinking test scores and intelligence test scores could have. If Models 1a or 2a fit best, we will interpret this to mean that the TTCT and ICAR measure separate but correlated constructs. If Model 3a fits best, then we will interpret this to indicate that the TTCT and ICAR measure a mix of verbal and non-verbal reasoning behaviors, whereas Model 4a being the best fitting model would support the view that TTCT tests measure different constructs and that the TTCT Figural test has a multifactorial structure. A best fitting Model 5 would indicate that all TTCT and ICAR tests are direct measures of *g* and that there are no separate constructs that these tests measure. If Models 1b or 2b fit the data best, then we will interpret this as indicating that the TTCT and ICAR measure separate first-order factors but that these then combine to form a second-order *g* factor. If Model 3b has the best fit indices, then this will mean that the tests measure a mix of verbal and non-verbal behaviors which then coalesce into a *g* factor. Finally, if Model 4b fits best, then this indicates that there is a general *g* factor among the subscores in the study, but that these subscores also form mediating factors for intelligence, verbal divergent thinking, an adaptive figural behavior, and innovative figural responses.

Finally, there is the possibility that no model will have adequate fit. If this occurs, then we will interpret this result to indicate that the factor structure of divergent thinking and intelligence subtest scores does not conform to any *a priori* theorized structure. The factor structure of these two tests in relation to each other will remain unresolved. We will not engage in any exploratory analyses (e.g., exploratory factor analysis, modification of confirmatory models to improve fit) if no pre-specified confirmatory models are found to fit the data.

### Predictions

We have no firm predictions about what this study may show, and we are agnostic about the results. To us, all nine of these models are plausible, and we do not have a strong belief about which may be the best fitting model. We also recognize that because fit statistics are sensitive to different aspects of the data and of model misspecification that we may get contradictory indications of what “the best fitting model” is. For example, a model could have the lowest BIC but higher SRMR and RMSEA values than other models. If contradictory results appear in the fit statistics, we will report this and interpret it to indicate that we could not fully resolve the question of how divergent thinking test scores and intelligence subtest scores interrelate but that we have narrowed the range of possibilities. There will be no attempts to modify models in order to improve fit.

### Open science practices

This project is designed to conform to all standards of open science. All methods and materials are now available to view at https://osf.io/8rpfz/?view_only=6413b521b5df4b8e877bc1e1c379d9c6. Publication as a Registered Report Protocol will function as a pre-registration for the study. After completion of the study, we will make all raw data, analysis syntax, and output files freely available online. We will also report any deviations from the pre-registered protocol and decisions made to handle any unforeseen events. We will also make a pre-print of the results available when submitting to the journal. After completion of the study, all data, syntax, and output files will be available to view and publicly download at https://osf.io/8rpfz. We will also make the response forms available to researchers who explain their qualifications and interests to us, which will permit future researchers to re-analyze our data with alternate scoring methods (such as those explained by Reiter-Palmon et al. [42]). Readers should note that making response booklets openly available online would violate copyright on the TTCT and item confidentiality of both tests, so we will control access to these materials.
